# Self medicated antibiotics in Bangladesh: a cross-sectional health survey conducted in the Rajshahi City

**DOI:** 10.1186/1471-2458-14-847

**Published:** 2014-08-14

**Authors:** Mohitosh Biswas, Manobendro Nath Roy, Md Imran Nur Manik, Md Shahid Hossain, SM Tafsirul Alam Tapu, Md Moniruzzaman, Sharmin Sultana

**Affiliations:** Department of Pharmacy, University of Rajshahi, Rajshahi, Bangladesh

**Keywords:** Self medication, Antibiotic, Rajshahi City, Bangladesh

## Abstract

**Background:**

Antibiotic self medication is highly prevalent in the developing countries due to easy availability and poor regulatory controls for selling these drugs. The purpose of this study was to evaluate the prevalence of self-medication with antibiotics for the treatment of various diseases by the peoples of Rajshahi city in Bangladesh.

**Methods:**

A cross-sectional survey was conducted to the patient’s (n = 1300) at eight locations of Rajshahi city in Bangladesh from March to April, 2014. The locations were selected by convenience and the study population within each study area was randomly selected. The survey was self-administered and included questions pertaining to self medicated drugs and antibiotic usage patterns as well. Data were analyzed using descriptive statistics.

**Results:**

It was found that 347 (26.69%) out of 1300 participants experienced self medication with antibiotics. Over fifty percent of the patients studied were between the ages of 21–30 years with 83.57% of them being males and 16.43% females. The highest percentage of self medicated antibiotics was metronidazole (50.43%) followed by azithromycin (20.75%), ciprofloxacin (11.53%), amoxicillin (10.37%) and tetracycline (7.49%) respectively. The key reasons for the self medication of antibiotics was the pre-experience (45.82%), suggestions from others (28.24%) and knowledgeable of the antibiotics (16.14%). The perceived symptoms to purchase the antibiotics independently was dysentery, diarrhea and food poisoning (36.02%), cold, cough and fever (28.24%), infection (12.97%), dental carries and toothache (9.22%), irritable bowel syndrome (3.46%), acne (4.32%), ear and throat pain (2.31%). The duration of maximum antibiotics usage was ranges between 0–10 years. Only 4.32% patient’s used self medicated antibiotics longer than 10 years. The patient’s compliance for self medication of antibiotics varies from excellent to no comments whereas only 6.92% patients reported side effects for the self medication of antibiotics.

**Conclusions:**

The results of this study confirm that antibiotic self-medication is a relatively frequent problem in Bangladesh. Drug Administration of Bangladesh should implement the regulatory controls immediately on the distribution and selling of antibiotics in order to reduce the frequency of antibiotic misuse.

## Background

Self medication is the use of drugs to treat self diagnosed disorders or symptoms, or the intermittent or continued use of a prescribed drug for chronic or recurrent disease or symptoms. The buyer diagnoses his own illness and buys a specific drug to treat it [1]. Although deleterious, self medication with antimicrobial compounds is an extremely ubiquitous practice in developing countries for reasons of cultural tradition, convenience, accessibility, perceived savings and other benefits, with poor adherence being an ever-present consequence of self medication [2]. The arsenal of antibiotics may be used up quickly, leaving the patient vulnerable to drug resistant infections. Treatment of bacterial infections in the hospital and community has been altered drastically over the past few decades with the emergence of pathogenic organisms that are no longer susceptible to our most commonly prescribed antibiotics. There is a clear relationship between the amount of a given antibiotic used and the incidence of bacterial resistance [3]. Resistance to commonly used antimicrobial drugs is remarkably high in countries where antibiotics are not restricted [4]. Increasing rates of antimicrobial resistance have left clinicians with limited drug options for the treatment of bacterial infectious diseases. This is a major public health concern worldwide, especially in developing countries where higher rates of resistant bacterial infections persist [5, 6]. Antibiotic resistance in developing countries causes a catastrophic increase in the medical and socio economic burden of untreatable infectious diseases [7].

Antimicrobial medication management in Bangladesh has been highlighted by previous studies as an area for improvement. Studies in Bangladesh, India, Thailand and Tanzania estimate that 24% to 50% of the total pharmaceutical budget is spent on antimicrobial agents [8–11]. These expenditures are much greater than those in West Germany or the United Kingdom, where expenditures for antimicrobial agents are 4% - 15% of the pharmaceutical costs [12]. There is already enough evidence of growing resistance to antimicrobials in Bangladesh resulting from misuse of antibiotics [13–15]. Because the misuse and abuse of antibiotics is a major cause of antimicrobial resistance, research is needed to evaluate the specific antibiotic usage patterns that are prevalent in developing countries so that interventions can be developed and implemented. For such interventions to be effective, we must also understand the underlying socio-cultural factors that contribute to antimicrobial misuse and the subsequent amplification of resistance in human populations. Previous study showed that antimicrobials are widely available (18% incidents) in the home medicine cabinets of the Dhaka City population [16]. Since there is no prescription-only drug in Bangladesh, people can purchase drugs like sedatives, antimicrobials without prescription even in the remote parts of the country [17, 18]. In Bangladesh, the prevalence of self medication is thought to be high, usually attributed to the fact that most drugs can be obtained from the pharmacies without prescription. As a result, minor illnesses are treated with antimicrobials which have a potential to harm the individual as well as the society at large. Studies on pattern of antibiotic self medication and its associated factors should be of interest to public health practitioners due to the dangers posed to the individual and society at large, more so in a country where the literacy level and regulation of drug use are on opposite ends of the spectrum.

This study was therefore designed to evaluate the pattern of antibiotic self medication amongst the common peoples of Rajshahi city located in the Northern region of the country.

## Methods

### Survey site

The study site was Rajshahi city situated in the north-west of Bangladesh and the divisional headquarters of Rajshahi Division as well as the administrative district that bears its name and is one of the seven metropolitan cities of Bangladesh having an estimated population of 853,000. Its total area is 96.69 km^2^ (37.33 sq mi) and is situated on the northern banks of the river Padma (or Ganges which is one of the major rivers of the Indian subcontinent). Rajshahi consists of 4 Thanas, 35 Wards and 175 Mahallahs [19]. Eight locations named Katakhali bazaar, Binodpur, Station Bazaar, Kazla, Talaimari, Vadhra, Shaheb bazaar and Laxmipur were selected for the collection of data because these sites are the main places of Rajshahi City.

### Study design and data collection

Patient’s-based, cross-sectional study was conducted in the Rajshahi city in March-April 2014. For this purpose, a self designed standard questionnaire was developed by the Principle investigator, Mohitosh Biswas, Lecturer of Pharmacy Department of Rajshahi University. The questionnaire contained some basic variables: Age and sex of patients, the types of antibiotics commonly purchased, the reasons for which the peoples engaged in antibiotic self medication, the self recognized symptoms for which the drugs were used, the duration of use of these drugs as well as the patient’s compliance regarding the self medication of antibiotics.

Twenty six (26) students of the Master of Pharmacy (M.Pharm) in the Department of Pharmacy of Rajshahi University were assigned and given instruction by the principle investigator for conducting this health survey. Each student was said to collect data from 50 respondents, therefore the sample size was 1300. Written consent was taken from each patient during this study. The data collectors were waiting in front of the pharmacy outlets and convinced the patient’s to participate in the interview session. Data were collected from the patients at any age group selected randomly by direct interviewing them who came to purchase the drugs from the pharmacies or use self medicated antibiotics for last three months.

### Data analysis

Each student analyzed the data individually and submitted the report to the principle investigator. The principle investigator then accumulated all the data. In this regard, descriptive statistics were applied to the collected data using Microsoft Excel software and results are finally expressed graphically in percentages.

### Ethical considerations

The study was conducted following the general principles (section 12) of WMA declaration of Helsinki [20]. This survey based research is also logistically supported by the Department of Pharmacy, University of Rajshahi. The human subjects involved in this study did not use any hazardous agents and samples were not collected from them. As the human subjects only participated in the interview, it was not a prerequisite to take any further approval from institutional ethics committee to conduct this survey based research.

## Results

It was found that 347 (26.69%) out of 1300 participants experienced self medication with antibiotics where 83.57% accounted for males and 16.43% females. The highest percentage of patient’s (56.48%) aged between 21–30 years purchased the antibiotics without prescription followed by 18.73% in the age group between 11–20 years and 12.68% in the age group between 31–40 years. The patient’s who were aged over 60 years (2.02%) purchased the least amount of antibiotics whereas the peoples aged between 0–10 years didn’t take any non-prescription antibiotics, Table 1.

**Figure 1:**
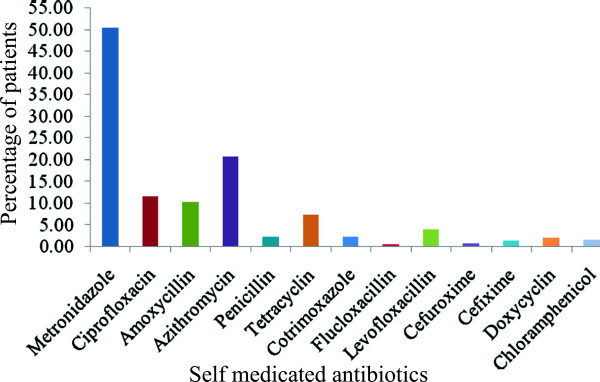
**Prevalence of self medicated antibiotics in the Rajshahi City of Bangladesh.**

**Figure 2:**
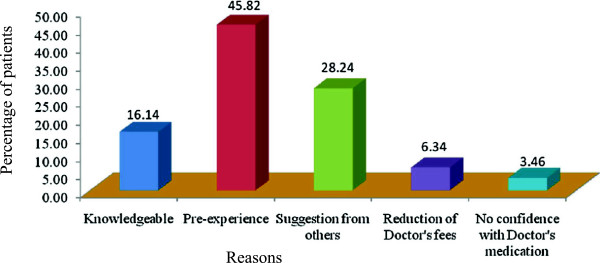
**Reasons for self medication of antibiotics.**

**Figure 3:**
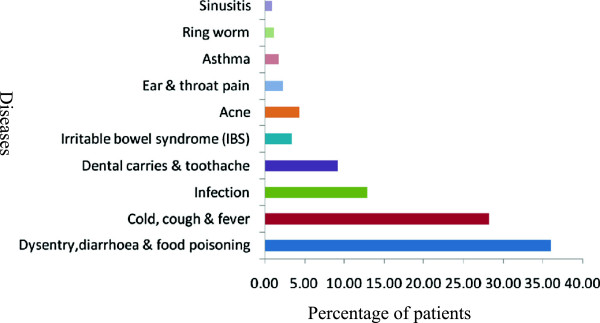
**Pathological reasons for self medication of antibiotics.**

The highest purchased self medicated antibiotics were metronidazole (50.43%) followed by azithromycin (20.75%), ciprofloxacin (11.53%), amoxicillin (10.37%) and tetracycline (7.49%). The antibiotics which were purchased in least percentage were flucloxacillin (0.58%) and cefuroxime (0.86%) shown in Figure 1. The key reasons for the self medication of antibiotics was the pre-experience (45.82%), suggestions from others (28.24%), knowledgeable of the antibiotics (16.14%), reduction of doctor’s fees (6.34%) and no confidence with doctor’s medication (3.46%), Figure 2. The main pathological factors that imposed the participants to purchase the antibiotics independently was dysentery, diarrhea and food poisoning (36.02%), cold, cough and fever (28.24%), infection (12.97%), dental carries and toothache (9.22%), irritable bowel syndrome (3.46%), acne (4.32%), ear and throat pain (2.31%), asthma (1.73%), ring worm (1.15%) and sinusitis (0.86%), Figure 3. Metronidazole was purchased to treat dysentery, diarrhea, food poisoning, dental carries, GI disturbance, bowel disorders, toothache, and protozoan infection. Azithromycin was self medicated to treat acne, irritable bowel syndrome, fever, cold, cough, tonsillitis and respiratory tract infections. Ciprofloxacin was taken for the ailment of irritable bowel syndrome, ear pain, fever, cold, cough, diarrhea, food poisoning, dysentery, abnormal digestion, urinary tract infection, respiratory tract infection, GI infection and disorders. Amoxicillin was taken for the treatment of ear pain, throat pain, fever, cough, common cold, dental carries, dental infection, anorexia, asthma and sinusitis. Tetracycline was self medicated for the treatment of cough, fever, acne, dysentery and diarrhea.

**Table 1:** **Prevalence of self medicated antibiotics and characteristics of the patients**

Question pattern	Response pattern	Total survey population N = 1300	Percentage (%)
Prevalence of self medication	Antibiotics	347	26.69
	Non-antibiotics	953	73.31
Age in Years of population (347) having self medicated antibiotics	0–10	0	0
	11–20	65	18.73
	21–30	196	56.45
	31–40	44	12.68
	41–50	24	6.92
	51–60	11	3.17
	>60	7	2.02
Gender of population (347) having self medicated antibiotics	Male	290	83.57
	Female	57	16.43

**Figure 4:**
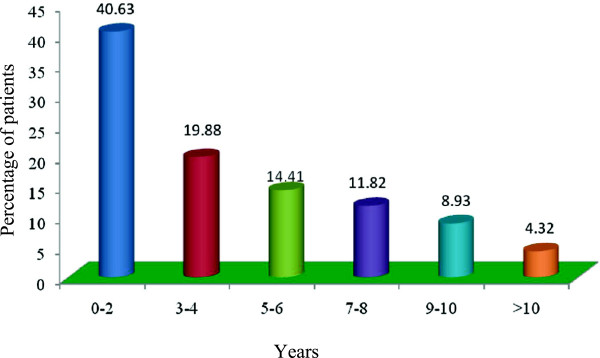
**Duration of self medication of antibiotics.**

**Figure 5:**
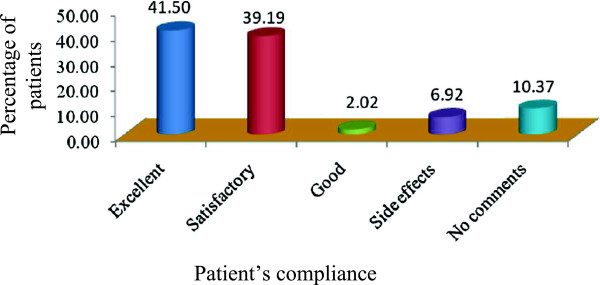
**Patient’s compliance for self medication of antibiotics.**

The duration of self medicated antibiotics usage vary greatly where 40.63% patients taking antibiotics by themselves for the duration of 0–2 years, 19.88% from 3–4 years, 14.41% from 5–6 years, 11.82% from 7–8 years, 8.93% from 9–10 years. Only 4.32% patient’s used self medicated antibiotics longer than 10 years, Figure 4. The patient’s compliance for self medication of antibiotics varies from excellent to no comments. Out of 347 patient’s, 41.50% reported that the disease recovery was excellent by taking the self medicated antibiotics, 39.19% was satisfactory for recovery of the diseases whereas disease recovery was good in 2.02% of the patients. Of them, 10.37% patients didn’t produce any comments regarding their compliance of antibiotic self medication but 6.92% patient’s reported side effects for the self medication of antibiotics, Figure 5.

## Discussion

This is the first formal study to report the self medication of antibiotics in the Rajshahi city of Bangladesh. Antibiotics were self medicated by 26.69% of the study population. This rate is fairly high compared with results conducted in six districts of Bangladesh (22.5%), India (18%), Jordan (23.0%), Viet Nam (12%) and Mongolia (21%) [21–25]. This is also in sharp contrast with reports from Turkey [26], where 19.1% prevalence was reported in antibiotic self medication among primary care attendants. In other studies in European countries, antimicrobial self medication were found to be much lower than 10% in each of 19 European countries spread across east, west, north and southern Europe [27]. These findings suggest how much needs to be done in tackling the issues of drug administration, distribution and control in developing countries including Bangladesh.

In our study, male (83.57%) tended to use more antibiotics than female which is concordance with the study conducted previously in Bangladesh but contrast in the study conducted in Lithuania [21, 28]. The high prevalence of self-medication was found within the adult participants (56.48%) which have correlation with the study conducted in Bangladesh and Arab Emirates [21, 29]. This could be explained by a number of factors, including the nature of the Bangladeshi peoples in which male dominates females in every spare of life even in self medication of antibiotics. This could also be explained by the fact that females are more cautious and sensible to their lives rather than males. Adults are independent enough to take their own decision in their life rather than pediatrics and geriatrics. This self dependency nature of adults also reflected in the self medication of antibiotics. No self medicated antibiotics were found in the age group between 0–10 years because they are dependent on their caregivers.

Metronidazole was the most commonly used antibiotic in this study which is concomitant with the similar results obtained in the previous study conducted in six districts of Bangladesh [21]. In a similar study in Mongolia, Arab Emirates and Nigeria [25, 29, 30], penicillins, particularly amoxicillin and ampicillin were more frequently used ahead of metronidazole for a variety of conditions by people who engaged in self medication. However, as the use of these agents is not guided by any biomedical knowledge, virtually any antibiotic could do for any ailment [31]. Diarrhea, dysentery and food poisoning were the symptoms for taking this antibiotic which reveals the poor hygienic food intake of the Bangladeshi peoples.

Of the non-prescribed antibiotics, past experiences and familiarity with a drug were the main reasons for selecting a particular antibiotic (45.82%). Many of our respondents used antibiotics because they considered themselves to be knowledgeable about antibiotic use (16.14%) based on their past experience. If antibiotics were previously prescribed for an infection and the later developed similar symptoms, then the users were more likely to use the same antibiotics. This reason runs counter to findings from other developing countries, where relatively lower costs have been given as the main reason for self-medication [22, 32] which is found 6.34% in our study. Studies from American, Asian and European countries indicate that between 22% and 70% of parents have misconceptions about the appropriate applications and efficacy of antibiotics and often use them without a prescription [24, 33, 34]. Other determinants of self-medication with antibiotics in low-income countries include over-the-counter sales of antibiotics, the high cost of medical consultations and dissatisfaction with medical practitioners [35]. In our survey based study, we have seen that 3.46% patient’s was antibiotic self medication because they had no confidence with the doctor’s medication.

Diarrhea, dysentery and food poisoning constitute the disease symptoms/conditions for which antibiotics were most commonly used in this study while 12.97% of responding patients used antibiotics for infections which include lower and upper respiratory tract infections, urinary tract infections, gastrointestinal infections, dental infections and skin infections. Acute respiratory infection was the condition associated most frequently with non-prescription antibiotic use, a result which substantiates findings from other Asian countries [24, 33]. Our results are also consistent with findings in China, where low-severity illness was a major reason for giving children antibiotics [33]. The finding of our study is also not much different from those of a similar study conducted in Bangladesh [21] and supports the assertion of Pradervant (1984), that in developing countries, antibiotics are viewed as wonder drugs capable of healing a wide variety of illnesses ranging from gastro intestinal disorders to headaches. As stated by the participants of our study, fever, cough and cold was the second major reason for treatment with self-medicated antibiotics. This finding, though consistent with results of other studies [23, 36, 37], also indicates the belief of the community that antibiotics can treat and eradicate any infections irrespective of their origin.

Although the non-prescription sale of antibiotics is illegal in Bangladesh, our results replicate findings from other studies in settings where pharmacies were the main source of antibiotics for self-medication [28, 33, 38]. In contrast, countries where over-the-counter antibiotics sales are strictly regulated have much lower prevalence rates of self-medication with antibiotics, ranging from 1% to 4% [27]. The widespread availability of antibiotics without a prescription has given rise to concerns about their increased usage [39]. The uncontrolled use of antibiotics can be harmful because of adverse drug reactions, masking of symptoms of infection, the development of chronic disease and super infection. It is also associated with the emergence and spread of antimicrobial resistance [40]. These problems require appropriate measures by policy-makers to develop pertinent policies as well as to ensure their implementation.

There are some limitations of our study. Since the participants were self-reporting via the questionnaire, we cannot be certain that we received all the relevant information related to their complaints and medicines (in terms of receiving or buying). Such bias may impact upon our results, but is difficult to avoid in questionnaire-based studies. The findings obtained from this small sample size (1300 only) cannot be generalized to the whole population of Bangladesh. To better study this issue, future research should focus on both urban and rural areas and should involve the patients as much as possible. Additionally, seasonal variations in illnesses should also be taken into consideration, because they may have impact on disease patterns and antibiotic usage. Despite these limitations, our findings have important significance in concern of current prevalence of self medicated antibiotics in Bangladesh that could help the drug regulatory authority of Bangladesh to implement restrictions for the distribution, selling and uses of antibiotic.

## Conclusions

Although antibiotics are prescription only drugs in Bangladesh, the results of this study confirm that antibiotic self-medication is a relatively frequent problem in the Bangladesh. It is the duty of the government especially Drug Administration of Bangladesh to implement the regulatory controls on the distribution and selling of antibiotics following the guidelines of National Drug Policy-2005 as well as to ensure the punishment for the persons who violate the regulations.

## Authors’ information

Mohitosh Biswas; Lecturer, Department of Pharmacy, University of Rajshahi, Rajshahi-6205, Bangladesh.

Manobendro Nath Roy, Md. Imran Nur Manik, Md. Shahid Hossain, S.M. Tafsirul Alam Tapu, Md. Moniruzzaman and Sharmin Sultana; M.Pharm Student, Department of Pharmacy, University of Rajshahi, Rajshahi-6205, Bangladesh.
